# Morphometric Analysis of Bone Lesions in a Patient With End-stage Renal Failure Due to Primary Hyperparathyroidism

**DOI:** 10.1210/jcemcr/luaf294

**Published:** 2025-12-18

**Authors:** Shoya Taniguchi, Tatsuya Suwabe, Kei Kono, Masayuki Yamanouchi, Naoki Sawa, Yoshifumi Ubara

**Affiliations:** Nephrology Center, Toranomon Hospital Kajigaya, Kanagawa 213-8587, Japan; Nephrology Center, Toranomon Hospital Kajigaya, Kanagawa 213-8587, Japan; Department of Pathology, Toranomon Hospital, Tokyo 105-8470, Japan; Nephrology Center, Toranomon Hospital Kajigaya, Kanagawa 213-8587, Japan; Nephrology Center, Toranomon Hospital Kajigaya, Kanagawa 213-8587, Japan; Nephrology Center, Toranomon Hospital Kajigaya, Kanagawa 213-8587, Japan

**Keywords:** primary hyperparathyroidism, bone biopsy, osteitis fibrosa, parathyroid hormone

## Abstract

A 59-year-old man presented for further evaluation of end-stage renal failure accompanied by hypercalcemia. For the past 10 years, his serum calcium levels had been above 10 mg/dL (SI: 2.49 mmol/L) (reference range, 8.7-10.1 mg/dL [SI: 2.17-2.52 mmol/L]) and he had experienced lower back pain and kyphosis of the thoracic spine. On admission, serum creatinine was 6.4 mg/dL (SI: 565.8 μmol/L) (reference range, 0.6-1.1 mg/dL [SI: 53-97 μmol/L]), but serum calcium was elevated 10.3 mg/dL (SI: 2.56 mmol/L) and intact PTH was significantly elevated 2837 pg/mL (SI: 301.0 pmol/L) (reference range, 10-65 pg/mL [SI:1.06-6.90 pmol/L]). Skull X-rays showed the salt and pepper sign and lumbar spine X-rays revealed the rugger jersey sign. A biopsy of the right ilium was performed. Cortical bone showed thinning associated with porosity, cancellous bone showed islanding due to bone loss, and the bone marrow had a high fat content. Some vigorous bone formation by osteoblasts was observed, but osteoclast-mediated bone resorption was predominant, suggesting osteoporosis with severe bone loss. Primary hyperparathyroidism was suspected as the underlying condition, so parathyroidectomy was performed. This case is a rare example of late diagnosis of primary hyperparathyroidism in a patient with end-stage renal failure and severe bone lesions.

## Introduction

Primary hyperparathyroidism is characterized by overproduction of PTH, which leads to elevated serum calcium levels. Prolonged hypercalcemia is well known to cause kidney stones and has been reported to be a cause of renal dysfunction. Few reports have described primary hyperparathyroidism that has progressed to end-stage renal failure requiring dialysis [1, 2], but here we report such a case.

## Case Presentation

**Figure 1.**
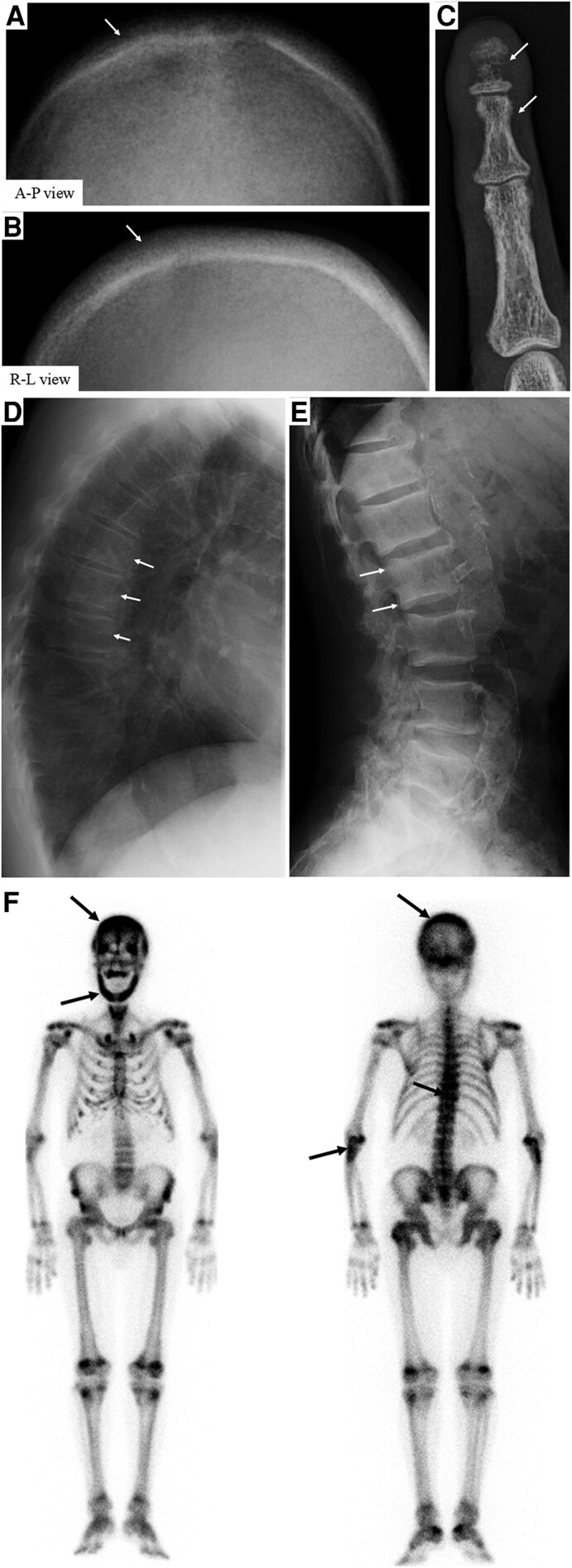
X-ray and bone scintigraphy findings. (A) Anteroposterior view, (B) right-left view. The X-ray examination showed the salt and pepper sign (multiple tiny well-defined lucencies caused by resorption of trabecular bone) in the skull. The margins of the skull were unclear. (C) Bone erosions and bone resorption (arrow) were noted in the second distal and intermediated phalangeal bones of the hand. (D) Kyphosis (arrow) of the thoracic spine was noted. (E) The lumbar spine showed the rugger jersey sign (defined by the sclerotic bands [arrows] on the inferior and superior endplates of the vertebral bodies and the alternating dense [arrow] to lucent-dense [arrow] appearance). (F) Bone scintigraphy showed a high uptake in the skull (arrow), thoracic and lumbar spine (arrows), and many joints (arrows).

A 59-year-old Japanese man was admitted to our hospital for evaluation of hypercalcemia and renal failure. The patient had been diagnosed with proteinuria and occult blood in urine at health checkups since he was in his 30s. Chronic glomerulonephritis such as IgA nephropathy was suspected, but because the amount of urinary protein was less than 0.5 g/day (reference range, < 0.15 g/day) and the urine occult blood test was only weakly positive, a kidney biopsy was not performed. At the age of 46 years, his serum creatinine rose to 2.2 mg/dL. However, given the slow progression of renal failure, the patient's doctor at the time suspected chronic glomerulonephritis (which progresses slowly) and treated the patient only with antihypertensive drugs such as calcium channel blockers. From around age 49 years, the patient’s serum calcium levels consistently exceeded 10 mg/dL (SI: 2.49 mmol/L) (reference range, 8.7-10.1 mg/dL [SI: 2.17-2.52 mmol/L]), and at around age 58 years, the patient noticed worsening thoracic kyphosis. At age 59 years, his serum creatinine level rose to 6.0 mg/dL (SI: 88 μmol/L) (reference range, 0.6-1.1 mg/dL [SI: 53-98 μmol/L]) and he experienced worsening chest and lumbar back pain. He also developed itching and irritability, so he visited our hospital to obtain a second opinion (Fig. 1).

On admission, the patient was 162 cm tall and weighed 62.3 kg. Noteworthy was that over the past 5 years, his height had decreased by 3 cm. Marked thoracolumbar kyphosis was noted.

The results of laboratory tests at admission were as follows: albumin, 3.2 mg/dL (SI: 32 g/L) (reference range, 3.9-5.2 g/dL [SI: 39.0-52.0 g/L]); creatinine, 6.4 mg/dL (SI: 565.8 μmol/L); estimated glomerular filtration rate, 7.9 mL/min/1.73 m^2^ (reference range, > 60 mL/min/1.73m²); calcium, 10.3 mg/dL (SI: 2.56 mmol/L); inorganic phosphate, 8.5 mg/dL (SI: 2.74 mmol/L) (reference range 2.8-4.6 mg/dL [SI: 1.2-1.5 mmol/L]) (Table 1).

**Table 1.** Laboratory tests at admission

Laboratory test	Case	Reference range
Serum		
Leukocytes	7100/μL	3400-9200/μL
Hemoglobin	8.1 g/dL (SI: 81 g/L)	13.0-17.0 g/dL (130-170 g/L)
Thrombocytes	16.8 ×10⁴/μL	14.1-32.7 ×10⁴/μL
AST	24 IU/L	13-30 IU/L
ALT	11 IU/L	10-42 IU/L
Total protein	6.3 g/dL (SI:63.0 g/dL)	6.9-8.4 g/dL (69.0-84.0 g/L)
Albumin	3.2 g/dL (SI: 32.0 g/L)	3.9-5.2 g/dL (39.0-52.0 g/L)
Uric nitrogen	129 mg/dL (SI: 46 mmol/L)	8-21 mg/dL (2.9-7.5 mmol/L)
Creatinine	6.4 mg/dL (SI: 565.8 μmol/L)	0.6-1.1 mg/dL (53-97 μmol/L)
Uric acid	9.1 mg/dL (SI: 541.2 μmol/L)	2.5-7.0 mg/dL (1487-416.8μmol/L)
Sodium	139 mmol/L	139-146 mmol/L
Potassium	4.3 mmol/L	3.7-4.8 mmol/L
Chloride	98 mmol/L	101-109 mmol/L
Calcium	10.3 mg/dL (SI: 2.57 mmol/L)	8.7-10.1 mg/dL (2.17-2.52 mmol/L)
Inorganic phosphate	8.5 mg/dL (SI: 2.74 mol/L)	2.8-4.6 mg/dL (1.2-1.5 mmol/L)
C-reactive protein	2.0 mg/dL (SI: 20 000 mmol/L)	<0.1 mg/dL (1000 mmol/L)
Glucose	90 mg/dL (SI: 5.0 mmol/L)	70-110 mg/dL (3.9-6.1 mmol/L)
Intact PTH	2837 pg/mL (SI: 301.0 pmol/L)	10-65 pg/mL (1.06-6.90 pmol/L)
PTHrP	<1.1 pmol/L (SI: <1.1 ng/L)	<1.1 pmol/L (<11 ng/L)
1,25 (OH)₂D	7.0 pg/mL (SI: 17.0 pmol/L)	20-60 pg/mL (48-144 pmol/L)
25(OH)₂D	5.2 ng/mL (SI: 13.0 pmol/L)	>30 ng/mL (74.9 pmol/L)
ALP	758 IU/L	117-350 IU/L
Bone-specific ALP	153.2 IU/L	9.6-35.4 IU/L
Osteocalcin	725 ng/mL (SI: 725 μg/L)	2.5-13 ng/mL (2.5-13 μg/L)
pH	7.39	
Bicarbonate (HCO³^−^)	23 mmol/L	22.2-28.3 mmol/L
eGFR	7.9 mL/min/1.73 m²	>60 mL/min/1.73 m²
Anion gap	18 mEq/L	10-14 mEq/L
Urine		
pH	5	
Protein	1.36 g/day	<0.15 g/day
NAG	12 IU/L	<11.5 IU/L
β₂-microglobulin	5884 μg/L	30-370 µg/L
Glucose	(−)	
Calcium	120.6 mg/day (SI: 30.1 mmol/day)	40-200 mg/day (9.98-49.9 mmol/day)
Phosphate	0.43 g/day (SI: 138.8 mmol/day)	0.5-0.9 g/day (161.5-290.6 mmol/day)
Aminoaciduria	(−)	
NTx/Cr	1585 nmol BCE/mmol·Cr	13.0-66.2 nmol BCE/mmol·Cr
Deoxypyridinoline	21.2 nmol/mmol·Cr	2.1-5.4 nmol/mmol·Cr
Tmp/GFR	0.52%	0.8-1.3%

Abbreviations: ALP, alkaline phosphatase; ALT, alanine aminotransferase; AST, aspartate aminotransferase; Cr, creatinine; eGFR, estimated glomerular filtration rate; intact PTH, intact PTH; NAG, alpha-N-acetylglucosaminidase; NTx, N-terminal telopeptide; PTHrP, parathyroid hormone-related peptide; Tmp/GFR, tubular maximum transport of phosphate per glomerular filtration rate.

In many kidney diseases, as kidney function declines, serum calcium levels typically decrease and serum phosphate and PTH levels increase. Although the patient had renal failure and was not receiving activated vitamin D3 (ie, 1,25(OH)_2_D_3_) or calcium preparations, he presented with hypercalcemia, so additional tests were performed. Albumin-adjusted serum calcium (calculated as serum calcium in mg/dL—albumin in g/dL + 4.0) was 11.1 mg/dL (SI: 2.76 mmol/L), and 1,25(OH)_2_D_3_ was 7.0 pg/mL (SI: 17.0 pmol/L) (reference range, 20-60 pg/mL [SI: 48-144 pmol/L]). The intact PTH level was 2837 pg/mL (SI: 301.0 pmol/L) (reference range, 10-65 pg/mL [SI: 1.06-6.90 pmol/L]); PTH-related protein, less than 1.1 pmol/L (SI: 11 ng/L) (reference range, < 11 pmol/L [SI: < 11 ng/L]); osteocalcin, 725 ng/mL (SI: 725 μg/L) (reference range, 2.5-13 ng/mL [SI: 2.5-13 μg/L]); alkaline phosphatase, 758 IU/L (reference range 117-350 IU/L); and bone alkaline phosphatase, 153.2 U/L (reference range, 9.8-35.4 IU/L).

**Figure 2.**
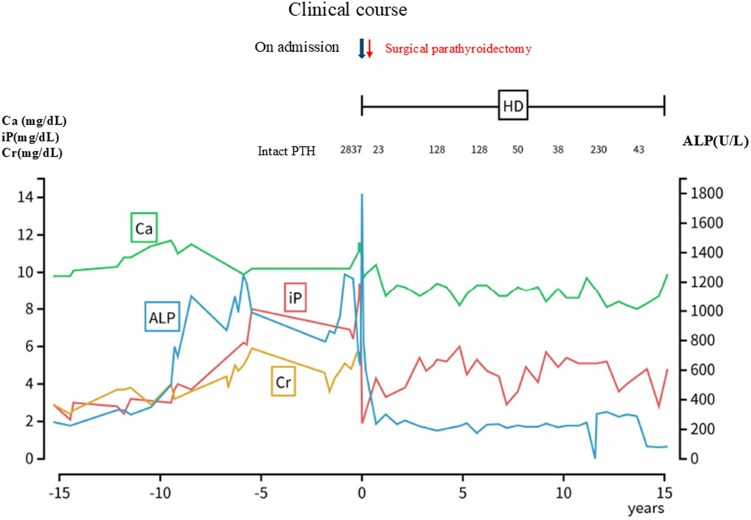
Clinical course. The figure shows changes in calcium, phosphate, intact PTH, and creatinine from before admission to after parathyroidectomy. ALP, alkaline phosphatase; Ca, serum calcium; Cr, serum creatinine; iPTH, intact PTH; iP, inorganic phosphate.

The X-ray examination showed the salt and pepper sign in the skull (Fig. 2A and 2B), bone erosions and bone resorption in the second phalangeal bone (Fig. 2C), kyphosis of the thoracic spine and deformation of the thorax (Fig. 2D), and the rugger jersey sign in the lumbar spine and severe calcification of the lumbar aorta (Fig. 2E).

Bone scintigraphy was performed with technetium 99m-labeled methylene diphosphonate to determine the causes such as osteomalacia, hyperparathyroidism, or metastatic bone tumors. It showed high uptake in the skull, thoracic and lumbar spine, and many joints (Fig. 2F). Dual X-ray absorptiometry showed bone mineral density T-scores of −3.5 and −1.5 in the right forearm (33% radius) and right hip (femoral neck), respectively.

As a result, a bone biopsy (the gold standard for confirming bone lesions) was performed to investigate these causes.

After obtaining informed consent from the patient, we examined his bone disease by performing double tetracycline (TC) labeling with doxycycline 200 mg/day (with a schedule of 3 days on, 7 days off, 3 days on, and 7 days off), and a right iliac crest bone biopsy was performed according to the previous methods [3-7].

**Figure 3.**
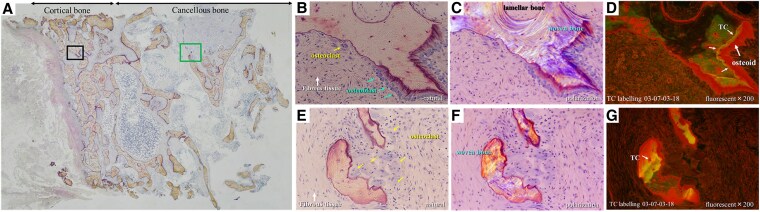
Crest bone biopsy findings. (A) Overall appearance of the crest bone biopsy. Thinning of cortical bone due to cortical bone porosity was prominent (original magnification × 20). (B-D) Enlarged view of the cortical bone in Fig. 3A (original magnification × 200). (B) Natural view: In cortical bone, active bone formation by osteoblasts was observed, but in other areas, bone resorption by osteoclasts was evident. Because of the extremely rapid bone resorption by multinucleated osteoclasts, the resorption of demineralized bone components was delayed, resulting in the retention of bone matrix as fibrous tissue (the amount of fibrous tissue serves as an indicator of bone turnover). (C) Polarization view: Lamellar bone formation (orderly arrangement) occurs through remodeling mechanisms, but in this case, active bone formation by osteoblasts resulted in disorganized woven bone formation (random arrangement) that did not match the load. (D) Fluorescent view: Double tetracycline (TC) labeling was clearly visible. The red areas outside the labeling represent premineralized osteoid. The presence of both double TC labeling and premineralized osteoid indicates rapid bone formation. (E-G) Enlarged view of the cancellous bone in Fig. 3A (original magnification × 200). (E) Natural view: In cancellous bone, bone trabeculae were reduced and island-like bones were prominent. Multinucleated osteoclasts were prominent around the bone. Furthermore, fibrous tissue that could not keep pace with bone resorption remained around the osteoclasts. Cone resorption was clearly more prevalent than bone formation. (F) Polarization view: Island-like bones did not appear to be lamellar bones but were mainly irregular woven bones (random arrangement). (G) Fluorescent view: TC markers indicating advanced bone formation were observed, with a large amount of red osteoid visible on the outer side. These findings suggest accelerated bone formation accompanied by delayed calcification.

Cortical bone showed thinning associated with porosity, cancellous bone showed islanding due to bone loss, and the bone marrow had a high fat content (Fig. 3A). Bone morphometry of the cancellous bone adjacent to the cortical bone was analyzed. The fibrous tissue volume to total volume was 8.37%, which was significantly higher than the reference value (<0.5%). The osteoid volume to total bone volume was 19.6%, which was also significantly higher than the reference value (>15%), and both the bone formation rate normalized by bone volume (316.6%/year; normal value, 16.2 ± 12.5%/year) and bone formation rate/bone surface (0.205 mm^3^/mm^2^/year; normal value, 0.010 ± 0.008 mm^3^/mm^2^/year) were increased (Table 2).

**Table 2.** Bone morphometry of the cancellous bone adjacent to the cortical bone

Parameter		Ratio or abbreviation	Measured value(iliac bone)	Reference range
Bone volume	Bone volume	BV/TV	15.6%	21.1 ± 3.2%
	Trabecular thickness	Tb.Th	129.3 μm	144.5 ± 17.1 μm
	Wall thickness	W.Th	NM	43.2 ± 2.9 μm
Osteoid	Osteoid volume	OV/TV	3.01%	0.1∼1.0%
	Osteoid volume	OV/BV	19.6%	4.9 ± 1.2%
	Osteoid surface	OS/BS	74.2%	23.2 ± 3.4%
	Osteoid thickness	O.Th	17.1 μm	11.6 ± 2.0 μm
	Osteoblast surface	Ob.S/BS	18.1%	
Resorption	Eroded surface	ES/BS	18.1%	5.6 ± 1.7%
	Osteoclast number	N.Oc/BS	1.32 N.mm	
	Fibrous volume	Fb.V/TV	8.37%	0%
Mineralization	Mineral apposition rate	MAR	1.49 μm/day	0.75 ± 0.14 μm/day
	Double labeled surface	dLS/BS²	17.3%	
	Single labeled surface	sLS/BS²	40.9%	
	Bone formation rate	BFR/BS²	0.205 mm³/mm²/year	0.01 ± 0.008 mm³/mm²/year
	Bone formation rate	BFR/BV²	316.6%/year	16.2 ± 12.5%/year

In cortical bone, we observed bone formation by osteoblasts, including woven bone formation; however, osteoclast-mediated bone resorption predominated, leading to a decrease in bone mass (Fig. 3B). In cancellous bone, osteoclast-mediated bone resorption exceeded osteoblast-mediated bone formation, resulting in a decrease in bone mass. Island-like bones were prominent (Fig. 3C). The patient was diagnosed with severe osteoporosis and osteitis fibrosa due to hyperparathyroidism. Double TC labeling was prominent in both cortical and cancellous bone, indicating that these were high turnover bones.

An ultrasound examination showed 4 parathyroid glands, of which the lower right gland was the largest (17 × 22 × 24 mm). Technetium-99 m methoxyisobutylisonitrile scintigraphy showed a positive image that corresponded to the largest parathyroid gland on ultrasound. Computed tomography showed only 1 small stone in the right kidney. Circumferential severe calcification was observed in the abdominal aorta, and calcification consistent with the mitral valve was also observed.

## Diagnostic Assessment

Primary hyperparathyroidism was also accompanied by bone lesions due to severe osteitis fibrosa with high turnover bone, prompting the decision to perform parathyroidectomy as the next treatment option.

## Treatment

During parathyroidectomy, all 4 parathyroid glands were removed, and a portion of the smallest parathyroid gland was transplanted subcutaneously into the left forearm. Immediately after the operation, hypocalcemia and tetany symptoms characteristic of hungry bone syndrome developed, necessitating temporary administration of large doses of active vitamin D preparations and calcium supplements.

The resected parathyroid specimen was examined. The largest parathyroid gland (ie, the one on the lower right) was a single adenoma weighing 6618 mg and showed necrosis and hemorrhage. The other 3 glands weighed 889, 666, and 435 mg and had a multinodular appearance, leading to a diagnosis of reactive hyperplasia. The enlargement of the largest gland was associated with primary hyperparathyroidism, whereas the enlargement of the other 3 glands was thought to reflect secondary hyperparathyroidism associated with long-term renal failure.

## Outcome and Follow-up

Postoperative intact PTH levels decreased to 9 pg/mL (SI: 0.96 pmol/L). However, renal failure progressed even after the surgery, and hemodialysis became necessary (Fig. 1). Immediately after surgery, the chest and lumbar back pain, itching, and irritability disappeared, and the progression of thoracic kyphosis stopped. No new fractures have been observed. The clinical course showed (Fig. 2).

In the 3 years after surgery, bone mineral density increased and was maintained during the subsequent 16 years.

## Discussion

Bone abnormalities commonly observed in patients undergoing long-term dialysis have been categorized as chronic kidney disease–mineral and bone disorders or renal osteodystrophy and include osteitis fibrosa, a well-known bone abnormality associated with secondary hyperparathyroidism [3-6]. In our patients, the presence of persistent hypercalcemia throughout the progression of renal failure was consistent with primary hyperparathyroidism. This is a rare case in which primary hyperparathyroidism was not diagnosed, and the patient progressed to end-stage renal failure. Renal complications of primary hyperparathyroidism include renal calculi, and renal dysfunction may occur [1, 2]. Several articles have reported on primary hyperparathyroidism and renal dysfunction. For example, Nair et al found that 30.4% of patients diagnosed with primary hyperparathyroidism had an estimated glomerular filtration rate below 60 mL/min/1.73 m^2^ [2]. A few articles have reported cases of primary hyperparathyroidism progressing to end-stage renal failure [8].

Furthermore, in the renal failure preservation phase (chronic kidney disease stage 5), our patient had bone lesions like those observed in long-term dialysis patients, emphasizing the importance of early diagnosis and appropriate treatment for this condition.

Some authors have described bone biopsy findings in patients with mild to moderate hyperparathyroidism [2, 8]. They reported that cortical bone thinning due to cortical bone porosity is considered characteristic but that the connectivity of trabecular bone in cancellous bone is maintained [9, 10].

In our case, we compared the patient's bone lesions with those previously reported in patients with primary hyperparathyroidism. Both cortical and cancellous bone showed high turnover with advanced bone formation. Interestingly, the thinning of cortical bone associated with porosity was consistent with previous reports, but the bone-loss phenomenon observed in cancellous bone was not. To interpret this inconsistency, we examined reports on bone lesions in patients with general osteoporosis or secondary hyperparathyroidism due to long-term dialysis; we discuss the findings next.

Hatano et al reported the characteristics of bone lesions in patients with rheumatoid arthritis who received long-term glucocorticoid therapy after menopause [7]. Cortical bone thinning and trabecular bone loss were characteristic, but a decrease in bone formation rate was also observed. The causes of bone loss were reported to include menopause, long-term glucocorticoid therapy, and immobilization.

Hatano et al [5] and Ubara et al [6] reported that differences in physical activity are related to the maintenance mechanism of cancellous bone [5, 6].

Upon reviewing the present case based on these reports, we realized that the patient's level of physical activity had significantly decreased over the past 5 years. Therefore, we assumed that in this patient, physical inactivity was related to the loss of cancellous bones.

Noteworthy is that after surgical treatment for primary hyperparathyroidism, the patient's activity level improved and bone density increased.

We investigated the pathology that led to end-stage renal failure in the present case. He was suspected to have primary glomerulonephritis. Furthermore, primary hyperparathyroidism was assumed to be a progressive factor.

In summary, this case highlights the importance of early diagnosis of primary hyperparathyroidism and the necessity of surgical and medical treatment because diagnostic delays can lead to the development of end-stage renal failure and severe bone lesions, as seen in this case.

## Learning Points

- In cases of persistent hypercalcemia, primary hyperparathyroidism should be considered in the differential diagnosis.
- Severe hyperparathyroidism can cause thinning of cortical bone associated with porosity.
- Decrease of physical activity is associated with loss of cancellous bone.
- Parathyroidectomy can be expected to improve bone density and activities of daily living.

## Contributors

All authors made individual contributions to authorship. S.T., T.S., K.K., M.Y., and N.S. were involved in the diagnosis and management of the patient. Y.U. was involved in genetic counseling for the patient and genetic test interpretation. All authors reviewed and approved the final draft.

## Data Availability

Original data generated and analyzed during this study are included in this published article.
